# Within patient microevolution of *Mycobacterium tuberculosis* correlates with heterogeneous responses to treatment

**DOI:** 10.1038/srep17507

**Published:** 2015-12-01

**Authors:** Qingyun Liu, Laura E. Via, Tao Luo, Lili Liang, Xin Liu, Sufang Wu, Qingyu Shen, Wang Wei, Xianglin Ruan, Xing Yuan, Guolong Zhang, Clifton E. Barry, Qian Gao

**Affiliations:** 1 Key Laboratory of Medical Molecular Virology of Ministries of Education and Health, Institutes of Biomedical Sciences and Institute of Medical Microbiology, School of Basic Medical Sciences, Fudan University, Shanghai 200032, China; 2 Tuberculosis Research Section, Laboratory of Clinical Infectious Diseases, NIAID, NIH, Bethesda, MD, 20892, USA; 3 Institute of Infectious Disease and Molecular Medicine, and the Department of Clinical Laboratory Sciences, Faculty of Health Sciences, University of Cape Town, Rondebosch 7701, Republic of South Africa; 4 Henan Provincial Chest Hospital, Zhengzhou 450003, Henan, China; 5 Sino-US International Research Center of Tuberculosis, ZhengZhou 450003, Henan, China

## Abstract

Genetic heterogeneity of *Mycobacterium tuberculosis* (MTB) within a patient has caused great concern as it might complicate antibiotic treatment and cause treatment failure. But the extent of genetic heterogeneity has not been described in detail nor has its association with heterogeneous treatment response. During treatment of a subject with MDR-TB, serial computed tomography (CT) scans showed this subject had six anatomically discrete lesions and they responded to treatment with disparate kinetics, suggesting heterogeneous MTB population may exist. To investigate this heterogeneity, we applied deep whole genome sequencing of serial sputum isolates and discovered that the MTB population within this patient contained three dominant sub-clones differing by 10 ~ 14 single nucleotide polymorphisms (SNPs). Differential mutation patterns in known resistance alleles indicated these sub-clones had different drug-resistance patterns, which may explain the heterogeneous treatment responses between lesions. Our results showed clear evidence of branched microevolution of MTB *in vivo*, which led to a diverse bacterial community. These findings indicated that complex sub-populations of MTB might coexist within patient and contribute to lesions’ disparate responses to antibiotic treatment.

It is now commonly accepted that the MTB population within individual tuberculosis (TB) patients can be more heterogeneous than was traditionally thought^1^,^2^,^3^. Heteroresistance, which refers to the coexistence of both drug-resistant and drug-sensitive strains or several drug-resistant strains with discrete drug resistance conferring mutations, has been described in clinical isolates of MTB previously^3^,^4^,^5^,^6^. In some cases, the genetic heterogeneity was due to mixed infections (polyclonal infections) by two distinct MTB strains with different drug susceptibilities that could be easily distinguished by standard fingerprinting^1^,^2^,^7^. In other cases, it was described as an intermediate or transitory state during the evolution of a single clonal strain to drug-resistance^3^,^6^,^8^. For instance, sequencing of serial isolates from a given patient revealed multiple resistance-conferring mutations that were transiently detected during development of drug resistance only one of which was ultimately fixed^3^,^9^,^10^. This phenomenon may have been the result of clonal interference because different drug-resistant mutations might result in different fitness costs^3^,^9^,^10^. An earlier study investigated different lesions obtained following lung surgery and found discrete pulmonary lesions carried strains with different drug-resistant mutations, which had evolved from a single parental strain^11^. This finding was further confirmed by analyzing the drug susceptibility profile of multiple cavity isolates from 5 patients that underwent pulmonary resection surgery^12^. In these situations, clonal interference does not apply because the strains reside in physically discrete lesions and genetic heteroresistance might therefore be a long-term phenomenon *in vivo*. In this study, taking advantage of the high resolution of deep whole genome sequencing together with molecular typing method, we tracked the population genetic dynamics of MTB population in serial sputum samples and characterized this phenomenon in more detail. Meanwhile, we provided the possible correlation between lesions’ disparate responses and heterogeneous bacterial population.

## Results

### Initial radiographic findings

**Figure 1:**
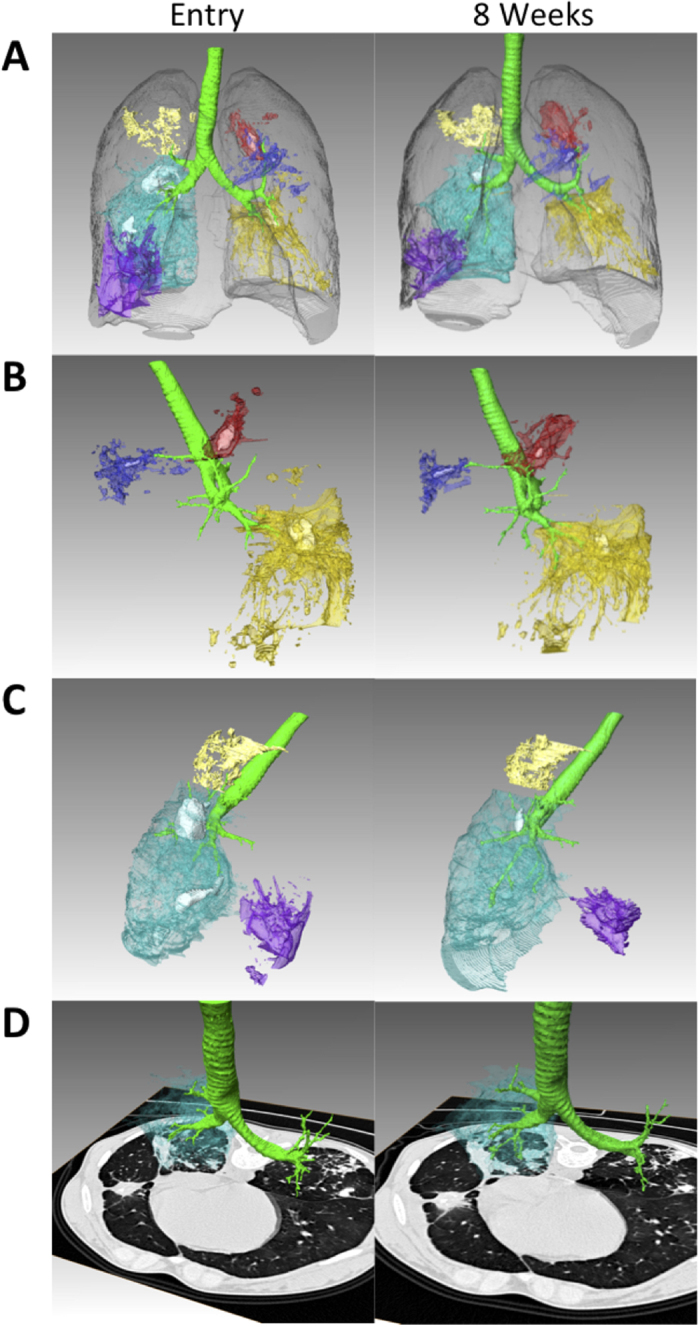
CT images from the subject at weeks 0 and 8. These images show six discrete lesion sites co-occurring in this subject. Each lesion is shown as a separate color and the extent represents “hard” lesion density (from −100 to + 200 Hounsfeld units) (PMID: 25473034). The left images show the results at study entry, prior to receiving TB chemotherapy and the result to the right show the same lesions after eight weeks of treatment. The airways are shown in green and the outline of the individual lungs is shown in transparent grey in the top images (**A**). In (**B)** the lesions contained within the left lung only are shown and the image is rotated to view this from the left side, likewise in (**C)** only lesions in the right lung are shown and the image is viewed from the right. In (**D)** the right lower lobe lesion, which increases 60% between the two scans is shown with an axial CT slice cut to show that the extent of disease does indeed increase. Rendering and quantification was performed with Amira 5.6.0 (FEI Visualization Sciences Group).

The subject described in this study had a previous history of tuberculosis and was thought to have been successfully treated as a child about 8 years ago. Upon presenting to the hospital with new signs and symptoms of tuberculosis, computed tomography (CT) images revealed bilateral disease with at least six spatially isolated lesions before treatment recommenced (Fig. 1—left panels). These six lesions included at least three moderate to large air pockets suggesting cavitary disease, all of which occurred at the distal end of a quaternary bronchus serving a distinct bronchopulmonary segment. Each of these was therefore potentially in direct contact with the airways and could be contributing bacilli to the sputum. In the left superior lobe there were two discrete apical lesions (red and blue in Fig. 1B), one of which did not appear cavitary. The posterior lesion (red), however, had a modest cavity containing 1.0 ml of air (white solid color in all lesions). The left inferior lobe was highly affected with a large lesion containing a central cavitation that contained 3.5 ml of air (lesion gold, air white in Fig. 1A,B). The right upper and middle lobes had non-cavitary lesions (tan and purple, respectively) but the right lower lobe had a large affected area with two separate air pockets occurring in discrete segments (light blue with two air pockets shown in white).

### Dramatic changes in bacillary load at week 6

**Figure 2:**
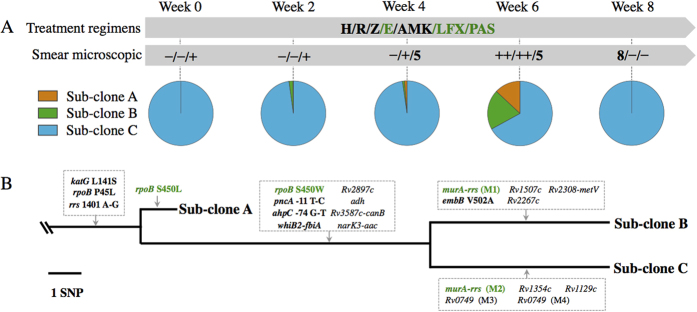
Treatment information of the subject and the trunk-branch genetic structure of the three sub-clones. (**A**) the first arrow shows treatment regimens, H, Isoniazid; R, Rifampicin; Z, Pyrazinamide; E, Ethambutol; AMK, Amikacin; LFX, Levofloxacin; PAS, Para-aminosalicylic acid. Drugs in green represent effective drugs. The second arrow contains the sputum smear microscopy results at each time point. ‘−’ represents smear negative; a number (such as 5, 8) represents the actual bacilli number that observed in 300 visual fields; ‘+’ represents 3 ~ 9 bacilli per 100 visual fields; ‘++’ represents 1 ~ 9 bacilli per 10 visual fields. The pie charts refer to the sub-clones identified through single colonies genotyping and the percentage of each chart represents the relative frequency of each sub-clone detected SNPs in deep sequencing data. (**B)** detailed phylogeny of the three sub-clones with common SNPs mapped to the trunk and heterogeneous SNPs mapped to the branches. Drug-resistant mutations are shown in bold and parallel mutations are highlighted in green. *murA-rrs* (M1) refers to SNP 1471660 G-C while *murA-rrs* (M2) refers to SNP 1471656 G-C. *Rv0749* (M3) and *Rv0749*(M4) refer to two different SNPs in *Rv0749*.

Considering the history of tuberculosis and the disease severity, the doctor prescribed an intensive treatment with seven anti-tuberculosis drugs (Fig. 2A). The drug susceptibility testing of this subjects’ MTB isolate at week 0 showed isoniazid and rifampin resistance making it formally multidrug-resistant (MDR-TB). In addition the isolate was also resistant to the injectable aminoglycoside amikacin making it “pre-extensively drug resistant” (pre-XDR). Thus although this subject received seven agents her isolate was only formally sensitive to ethambutol (E), levofloxacin (LFX) and *para*-amino salicylic acid (PAS). During the treatment, the sputum bacillary load as indicated by sputum smear score was low from week 0 to week 4 but showed a dramatic increase at week 6 (Fig. 2A). Then, sputum bacillary load decreased at week 8 under the same treatment regimens.

### Disparate treatment response by radiography

This subject received a repeat CT examination at eight weeks after starting TB chemotherapy. The lesions in this subject did not respond homogeneously to treatment (Fig. 1, right panels and Table 1). For example the lesion in the anterior segment of the left superior lobe showed a 42% decrease in the amount of abnormal density, while the two other lesions in her left lung showed modest increases in abnormal density, despite significant declines in the amount of cavitary air present in both lesions. In the right lower lobe her largest lesion showed a substantial increase in abnormal density at eight weeks (see detail in Fig. 1D, the axial section shows that although the extent of disease did not change that much, the amount of radiodense material inside the lesion increased substantially), despite this the nearby middle lobe lesion (purple) showed a significant decline in radiodense material (36%). This mixed response suggests that individual lesions might contain different sub-populations of bacteria with differential drug sensitivities.

### Deep sequencing revealed mixed MTB sub-populations

Several factors could contribute to this mixed response such as genetic heterogeneity of bacteria, presence of cavities, prior scars and tissue penetration of the drugs, etc. To uncover whether genetic heterogeneity of MTB population existed within this patient, we applied deep whole genome sequencing of serial initial cultures from week 0 to week 8. Compared with the reference genome for CCDC5079, there were 563 SNPs at weeks 0, 2, 4, 8 but this number changed to 552 at week 6. At week 6, the 552 SNPs were wholly within the 563 SNPs, but the other 11 SNPs were not consistently present in the MTB population occurring in between 66 ~ 87% of total reads (Table S1). This suggested that the strain carrying the 563 SNPs was still present at week 6, but that it was mixed with some other sub-clones that showed discrete sequences at 11 other loci. Meanwhile, several new SNPs increased in the population with the frequency of 13 ~ 21%, which was a complementary frequency to that of the 11 SNPs that were not fully penetrant and suggested they were carried by the new emerging sub-clones (Table S1).

### Verification of SNPs present in sub-clones

To verify that these results were due to mixed sub-populations, we re-cultured stored sputum sediment from week 6 and selected 25 single colonies for SNP genotyping. Three different sub-clones were identified with different mutation patterns that showing differential drug-resistant spectrum (Table S2). Sub-clone A carried 4 drug-resistant mutations (resistance to 3 drugs, RIF/INH/AMK), sub-clone B 7 drug-resistant mutations (resistance to 5 drugs, RIF/INH/EMB/PZA/AMK), and sub-clone C 6 drug-resistant mutations (resistance to 4 drugs, RIF/INH/PZA/AMK). An additional 14 non-resistance conferring or predicted resistance associated mutations (*whiB2-fbiA* and *murA-rrs*) were detected in 12 genes/regions, which could be either hitchhiking or compensatory mutations. These results provided a possible explanation for the disparate treatment response of different lesions that MTB sub-populations reacted differentially to the drug treatment. Based on the sub-clone specific SNPs, we estimated the percentage of the three sub-clones at different time points through deep sequencing data (Fig. 2A). Only sub-clone C was detected at week 0, sub-clone A and B appeared at a very low frequency at week 2 and 4. Only sub-clone C was detected in the week 8 culture. The lack of concurrence of the three sub-clones at weeks 0 and 8 suggests these bacterial sub-populations might emerge into the sputum from discrete lesions.

### Branched microevolution of MTB within patient

As all other 552 SNPs were still presented with frequency of 100% at week 6, the only difference between the three sub-clones were the 19 SNPs identified above (Table S2). Molecular typing of the three sub-clones showed consistent VNTR pattern across all the 25 single colonies (Table S3), suggesting they evolved from the same parental strain. Based on the mutational differences, we constructed the phylogeny of the three sub-clones and mapped their common drug-resistant mutations to the root (Fig. 2B). Large genetic distances were accumulated between the sub-clones with 14 SNPs between sub-clones A and B, 14 SNPs between A and C, 10 SNPs between B and C. All the three sub-clones carried mutations of *katG* L141S, *rpoB* P45L, *rrs* 1401 A-G. Mutation *rpoB* P45L might be associated with low-level RIF-resistance. Then, sub-clone A first diverged and accumulated a high-level RIF-resistant mutation *rpoB* S450L. The parent strain of sub-clones B and C accumulated another parallel high-level RIF-resistant mutation *rpoB* S450W, PZA-resistant mutation (*pncA* promoter −11 T-C) and INH-compensatory mutation (*ahpC* promoter −74 G-T). Then, sub-clone B acquired an EMB-resistant mutation *embB* V502A and a mutation in *murA-rrs* intergenic region, while sub-clone C acquired another mutation also in *murA-rrs* intergenic region, which was predicted to be associated with drug resistance^13^,^14^. These results demonstrated that branched microevolution of MTB within patient could lead to a diverse community and might be responsible for the disparate treatment responses of lesions.

## Discussion

In this study, we studied in detail the radiologic response of a single patient with extensive, bilateral TB and showed that the response to treatment in individual lesions was disparate based upon radiologic changes. By deep whole genome sequencing we were able to trace the genetic dynamics of MTB within this subject during treatment. There were three individual cavitary lesions in direct communication with the airways and we identified three branched-evolved sub-clones in this patient with a genetic distance between them of 10 ~ 14 SNPs. Although the three sub-clones were only simultaneously detected with comparable percentages at week 6, they were still likely present throughout the treatment but may have not been identified at the same time due to several potential factors such as limitations imposed by sputum sampling approaches, different degrees of cavitation and communication with the bronchi, etc. A likely explanation for this observation is that the sub-clones resided in different lesions and that sub-clones A and B gained access to the airways as treatment progressed. This hypothesis is supported by the coincidentally observed increase of bacilli load in sputum at week 6 when the three sub-clones were detected simultaneously and previous observations of lesions containing heterogenic populations^11^,^15^.

There are two possible explanations for the coexistence of the three sub-clones in a single individual. One is microevolution of a single clonal founder strain *in vivo* and another is mixed infections/re-infection. We think the former is more plausible based on the following reasons. First, in the previously reported polyclonal mixed infections/re-infection cases, the strains that infected the same patient showed distinct molecular typing patterns or differed in hundreds of SNPs^16^,^17^,^18^,^19^,^20^. Here, all three sub-clones showed a consistent VNTR pattern and the number of SNPs between them were very small, near the limits of the definition accepted for epidemiologic linkage^21^,^22^. Second, the mutations between the three sub-clones were mostly drug-resistant mutations, suggesting that they emerged under drug selective pressure. The parallel mutation patterns between them (*rpoB* mutations and mutations in *murA-rrs* intergenic region) further suggest the selective pressures on them were quite similar. Thus, considering this patient had a TB history, we speculate the three sub-clones evolved in parallel during the last treatment episode and persisted for years till relapse.

As sub-clone A and B had actually diverged before sub-clone C and sub-clone C was firstly detected at week 0, we can infer the three sub-clones already existed before treatment started. The last treatment of this subject was more than 8 years ago and we speculate a highly heterogeneous population persisted within the patient after childhood treatment was completed. This case of branched microevolution, and the few others that have been documented^3^,^8^,^9^,^11^, extends our understanding that adaptive evolution of MTB *in vivo* might be frequent rather than rare. Tens of MTB genomic regions were newly identified to be under positive selection in drug-resistant strains as the related mutations arise multiple times independently^14^. Among them, mutations in *murA-rrs* intergenic region (annotated as *rrs* promoter) were statistically associated with drug resistance^14^. In this study, the two different *murA-rrs* mutations identified in sub-clones B and C actually located in the coding sequence of a small regulatory RNA mcr3^23^. The accumulation of an independent mutation in each of the sub-clones B and C strongly suggests this regulatory RNA is involved in drug resistance/compensatory evolution.

Historically, five or fewer SNPs have been considered as a threshold to determine epidemiologically clustered strains, and pairs that differed by 6 to 12 SNPs were thought to be indeterminate for linkages^22^. However, a recent study showed 2 respiratory clonal variants from a single patient were separated by 7 SNPs and concluded genetic diversity within a patient can be as high as that found between patients^24^. Here, we found the genetic distance between sub-clones in a single individual was up to 14 SNPs, which supports the argument by Perez-Lago *et al*. and further reinforces the argument that this phenomenon might not be rare in clinic. Molecular genotyping of the three sub-clones showed the same VNTR pattern, supporting our conclusion that the three sub-clones found in this subject were not due to mixed infections despite the 14 SNPs genetic distance. More recently, phenotypic heteroresistance among HIV-infected patients who are being treated for MDR-TB has been associated with poor outcomes and longer time to culture conversion^25^,^26^. Therefore, understanding of genetic dynamics of MTB *in vivo* would provide us valuable information for adjusting treatment regimens.

Over the last 3 years, an unprecedented number of publications focusing on the intratumor genetic heterogeneity revealed that branched evolution was ubiquitous during progression of all tumors^27^,^28^,^29^,^30^. These findings suggest that tumor cells should be viewed as complex ecosystems, in which the growth of entire tumor may be influenced by a minor tumor sub-population and would complicate either biomarker development or a personalized therapy strategy^31^. To some extent, evolution of tumor cells and pathogenic bacteria are quite analogous as they share many common features including asexually clonal population structure, expanding, metastases, and the evolution of drug resistance^32^. Like tumor metastases, multiple lesions could rise during the development of chronic infection with MTB and they have a big range of dynamics from complete resolution to significant progression^33^,^34^,^35^. Thus, the MTB evolution within patient might not be always in a linear path but may show more diverse outcomes. Just as demonstrated in this study, we would have missed the other two dominant sub-clones if we had only taken sputum samples at week 0. Sequencing multiple sputum samples (from different time points early during treatment) may reveal sub-populations with cryptic drug resistance and allow for a faster switch to more appropriate treatment regimens.

However, our study still has several limitations: 1) Although the observed differential drug susceptibilities of sub-clones might be a possible explanation for the disparate responses of different lesions, there are still some other factors that could contribute to this phenomenon such as the presence of cavities, prior scars, proximity to bronchi, tissue penetration of the drugs, etc. 2) Given that the detection of MTB sub-populations were from pooled sputum samples (possibly with bacilli from all lesions) and not surgically resected samples, it is not possible to directly determine whether there was a differential distribution of sub-populations in different lesions. It therefore remains possible that the microevolution of MTB within the host took place in a single cavitary lesion.

In conclusion, we provided a detail depiction of the complexity of MTB population *in vivo*, which might contribute to the disparate treatment response observed by radiography. The remarkable genetic heterogeneity between the sub-clones provides interesting clues about the molecular events during the evolution of drug-resistance and highlights that branched microevolution of MTB within patient can lead to a diverse community.

## Methods

### Ethics and Patient information

The study to investigate the range of tuberculosis presentation and treatment (NCT01071603, clinicaltrials.gov) conducted in Henan Provincial Chest Hospital (HPCH) was approved by the HPCH and National Institute of Allergy and Infectious Diseases institutional review boards and subjects gave written consent for the study. The methods of this study were carried out in accordance with the approved guidelines and written informed consent was obtained from the subjects prior to the study. The female subject in this study was 22 years old when she enrolled in the study and reported that she had been treated for tuberculosis 8 years earlier and that she neither smoked tobacco nor consumed alcohol. Intensive treatment with 7 drugs (HRZE+AMK/LFX/PAS) was prescribed from week 0 to week 8. Drug susceptibility testing (DST) of the initial isolate showed resistance to INH/RIF/SM/AMK but susceptibility to EMB. From week 8, rifapentine (RPT) was added into the treatment regimen after the DST report became available.

### Sample collection and genome sequencing

We collected serial sputum samples from TB patients at 7 time points (week 0, week 2, week 4, week 6, week 8, week 16 and week 24). For each time point, three sputum samples were collected. Sputum smear microscopy, L-J culture and MGIT culture were preformed from each sputum sample. At each time point, if culture positive, we selected one L-J culture for whole population deep sequencing. That is, we scraped all the colonies on L-J medium and extracted genome DNA through CTAB method described before^3^. Whole genome sequencing was performed on Illumina Hiseq 2000 platform and the average sequencing depth was ~1000 folds. Sequencing reads have been submitted to the NCBI Sequence Read Archive (SRA) under accession code SRP057886.

### SNP calling and Sub-clones identification

A pipeline used for calling fixed/unfixed SNPs was previously introduced^3^. Simply put, *scythe* (https://github.com/ucdavis-bioinformatics/scythe) and *sickle* (https://github.com/ucdavis-bioinformatics/sickle) were used for trimming reads, *bwa*^36^ was used for mapping and *samtools* was used for SNP calling. The reference genomes H37Rv (GenBank accession NC_000962.3) and CCDC5079 (GenBank accession NC_021251.1) were used as templates for variants calling. For unfixed SNPs calling, we additionally used *lofreq*^37^ and *varscan2*^38^ besides the pipeline we used before^3^. The unfixed SNPs that were consistently detected by these methods and independent from the template we used were taken into consideration. For sub-clone validations, another stored sputum sample from week 6 was cultured on L-J plate. We selected 25 single colonies and extracted DNA through boiling. Primers were designed to amplify the fragments where the 19 SNPs we identified with frequency at week 6 located. Sanger sequencing was performed to detect the existence of each SNP. We used a well-characterized 12-loci VNTR (Variable-Number Tandem Repeat) genotyping method for the molecular typing of the 25 single colonies^39^.

## Additional Information

**How to cite this article**: Liu, Q. *et al*. Within patient microevolution of *Mycobacterium tuberculosis* correlates with heterogeneous responses to treatment. *Sci. Rep.* **5**, 17507; doi: 10.1038/srep17507 (2015).

## Supplementary Material

Supplementary Information

## Figures and Tables

**Table 1:** Radiographic characteristics of individual lesions.

**Lesion**	*Volume—Initial	**Volume—2 Mo**	**% Change**
Left Superior—Apical, Posterior (Red)	5.7	11.5	+50
Left Superior—Apical, Anterior (Blue)	4.4	3.1	−42
Left Inferior—Subcarinal (Gold)	32.3	40.6	+20
Right Upper—Apical, Posterior (Tan)	4.0	6.3	+58
Right Middle—Anterior (Purple)	18.4	11.8	−36
Right Lower—Posterior (Light Blue)	148	236	+60
**Air**	**Volume—Initial	**Volume**—**2 Mo**	**% Change**
Left Superior—Apical, Posterior (Red)	1.0	0.1	−90
Left Inferior—Subcarinal (Gold)	3.5	0.7	−80
Right Lower—Posterior (Light Blue)	13.5	0.6	−96

^*^Volume refers to the amount of radiodense (−100 to + 200 Hounsfeld Units) tissue in the indicated region in mL. **Air Volume refers to the amount of radiolucent space interior to a cavity (−1050 to −900 Hounsfeld Units).
